# Magnocurarine inhibits migration and invasion by suppressing mesenchymal marker expression and MAPK signaling in castration-resistant prostate cancer cells

**DOI:** 10.3389/fphar.2026.1903084

**Published:** 2026-08-31

**Authors:** Warathit Semmarath, Punnida Arjsri, Kamonwan Srisawad, Pilaiporn Thippraphan, Intranee Intanil, Sonthaya Umsumarng, Pornngarm Dejkriengkraikul

**Affiliations:** 1 Akkhraratchakumari Veterinary College, Walailak University, Nakhon Si Thammarat, Thailand; 2 Centre for One Health, Walailak University, Nakhon Si Thammarat, Thailand; 3 Department of Biochemistry, Faculty of Medicine, Chiang Mai University, Chiang Mai, Thailand; 4 Anticarcinogenesis and Apoptosis Research Cluster, Faculty of Medicine, Chiang Mai University, Chiang Mai, Thailand; 5 Faculty of Veterinary Medicine, Chiang Mai University, Chiang Mai, Thailand

**Keywords:** cancer cell invasion, castration-resistant prostate cancer, epithelial–mesenchymal transition, magnocurarine, MAPK signaling, phytochemical alkaloid

## Abstract

**Background:** Magnocurarine is an alkaloid isolated from several traditional Asian medicinal plants, including Magnolia and Harrisonia species. Previous studies have reported its anti-nociceptive, anti-inflammatory, and antimicrobial activities with relatively low cytotoxicity. However, the anticancer activity of magnocurarine, particularly its effects on prostate cancer cell migration and invasion, has not been well characterized. **Objective:** This study aims to investigate the effects of magnocurarine on the migration and invasion of castration-resistant prostate cancer (CRPC) cells. **Methods:** DU145 and PC3 cells were treated with non-toxic concentrations of magnocurarine (0-40 µM), and migration and invasion capabilities were evaluated using scratch wound healing and transwell invasion assays, gelatin zymography, and western blot analysis. **Results:** Magnocurarine significantly reduced the migration and invasion of CRPC cells in a dose-dependent manner (p < 0.05). The inhibitory effects were associated with decreased activities of MMP-2 and MMP-9, together with reduced expression of invasion-related proteins including uPA, uPAR, and MT1-MMP. In addition, magnocurarine suppressed the expression of mesenchymal markers, including N-cadherin, vimentin, and fibronectin. Further investigation showed that magnocurarine decreased the phosphorylation of ERK, JNK, and p38 without altering total protein expression, suggesting suppression of MAPK signaling activation. **Conclusion:** These findings indicate that magnocurarine attenuates migratory and invasive phenotypes in CRPC cells and is associated with reduced activation of MAPK signaling. This study provides initial evidence supporting the anti-migratory and anti-invasive effects of magnocurarine in prostate cancer cells and suggests its potential for further investigation as a phytochemical-derived drug candidate in advanced prostate cancer.

## 1 Introduction

Prostate cancer is one of the most common malignancies among men worldwide and remains a major global health concern (Raychaudhuri et al., 2025; Schafer et al., 2025). Although many prostate cancers are relatively indolent and confined to the prostate gland, a subset of patients develops aggressive disease requiring therapeutic intervention (Cheng et al., 2026). Current treatment strategies include prostatectomy and androgen-deprivation therapy (ADT), which targets androgen receptor signaling to suppress tumor growth (Choi et al., 2022; Raval et al., 2025). Although androgen-deprivation therapy is initially effective in many patients, disease progression to castration-resistant prostate cancer (CRPC) frequently occurs (Chandrasekar et al., 2015; Zhang et al., 2025).

CRPC exhibits aggressive biological features, including enhanced cellular proliferation, increased invasiveness, and a high metastatic potential, all of which contribute to poor prognosis and reduced overall survival (Muniyan et al., 2022; Kato et al., 2025). In particular, metastatic castration-resistant prostate cancer (mCRPC) remains largely incurable due to the limited efficacy of existing therapeutic options (Garje et al., 2025). Chemotherapeutic agents such as docetaxel and cabazitaxel are commonly employed as first-line treatments and can improve patient outcomes (Jha et al., 2014; Lăzescu et al., 2025). However, drug resistance and treatment-related adverse effects remain major clinical challenges, highlighting the need for alternative therapeutic strategies (Grewal et al., 2025).

At the molecular level, CRPC progression is closely associated with the dysregulation of multiple intracellular signaling pathways that govern tumor growth, invasion, and metastasis (Fang et al., 2025; Wu et al., 2025). Among these, the mitogen-activated protein kinase (MAPK) signaling pathway has been identified as a critical regulator of prostate cancer progression (Mukherjee et al., 2011; Cheung et al., 2021; Okorochi et al., 2025). MAPK activation has been associated with increased cell proliferation, EMT, migration, and invasion in prostate cancer cells, thereby contributing to metastatic progression (Inoue and Ogawa, 2011; Inan and Hayran, 2019; Xu et al., 2025). Furthermore, MAPK signaling interacts with androgen receptor signaling and other oncogenic pathways, contributing to therapeutic resistance and the development of the castration-resistant phenotype (Nickols et al., 2019; Bahmad, 2023). Together, these findings suggest that MAPK signaling may represent a therapeutic target in CRPC.

In recent years, bioactive compounds derived from natural products have attracted considerable attention for their ability to modulate oncogenic signaling pathways with relatively low toxicity. Recent studies have illustrated this potential in prostate cancer, in which Chinese herbal medicines and their constituent natural compounds, including flavonoids, terpenes, phenols, quinones, have demonstrated therapeutic potential by targeting the androgen receptor (AR), a central driver of prostate cancer development and progression, including CRPC cell models (Wu et al., 2024). Several herbal medicine interventions have shown promising results in inhibiting the expression of AR, inducing apoptosis, suppressing the proliferation of prostate cancer cells, and reducing the migratory and invasive potential of prostate cancer cells. For example, icaritin, extracted from *Epimedium brevicornu*, targets cyclin-related proteins and suppresses cell invasion by modulating the PEA3/HER2/AR signaling pathway in LNCaP cells (Hu et al., 2016). Bakuchiol, derived from *Psoralea corylifolia*, inactivated NF-κB signaling through modulation of AR and estrogen receptor beta (Erβ), thereby inhibiting the proliferation and migration of PC-3 prostate cancer cells (Miao et al., 2018). Beyond their direct antitumor and anticancer progression effects, naturally occurring compounds can be considered as adjuvant therapy in integrated comprehensive treatment plan that includes conventional medical treatments, such as surgery, radiation therapy, chemotherapy, and hormone therapy emphasizing the broader therapeutic potential of natural compounds in castration-resistant prostate cancers.

Magnocurarine is a water-soluble alkaloid isolated from Magnoliaceous plants, including Magnolia obovata and Magnolia officinalis, and is also found in *Harrisonia abyssinica* and *Harrisonia perforata*, which are used in traditional Asian and Thai medicine (Yan et al., 2013; Juckmeta et al., 2014). Previous studies have demonstrated that magnocurarine possesses diverse pharmacological activities, including anti-nociceptive, antioxidant, anti-diabetic, and anti-inflammatory effects (Zhao et al., 2006; Patil et al., 2010; Street et al., 2017). However, despite these reported biological properties, its role in cancer progression, particularly in the regulation of metastatic behavior, has not been clearly elucidated.

Therefore, in this study, the inhibitory effects of magnocurarine on CRPC cancer cells progression and metastatic potentials have been investigated in DU145 and PC3 prostate cancer cell lines. Specifically, we evaluated its effects on cell migration, invasion, and metastasis-associated protein expression, with a particular focus on the modulation of MAPK signaling pathways. This study aimed to investigate the effects of magnocurarine on migration, invasion, and metastasis-related protein expression in CRPC cells, with particular emphasis on MAPK signaling modulation. The findings may improve understanding of the anti-metastatic potential of magnocurarine in advanced prostate cancer drug development.

## 2 Materials and methods

### 2.1 Ethical statement

This study was approved by the Institutional Biosafety Committee–Chiang Mai University (CMU-IBC) (CMUIBC02019/2569).

### 2.2 Cell culture

The prostate cancer cell lines, DU145 and PC3 cells, were obtained from American Type Culture Collection (ATCC). The cells were cultured in RPMI-1640 medium (Sigma-Aldrich, St. Louis, MO, United States) supplemented with 10% fetal bovine serum (FBS), 2 mM L-glutamine, 50 U/mL penicillin, and 50 μg/mL streptomycin at 37 °C in a humidified atmosphere containing 5% CO_2_.

Primary human dermal fibroblasts were aseptically isolated from an abdominal scar following a cesarean delivery. The study was conducted under the ethical approval of the Medical Research Ethics Committee, Chiang Mai University (Study code: BIO-2567-0035). Fibroblast cells were isolated using a previously described protocol (Mapoung et al., 2020).

### 2.3 Cytotoxicity assessment

The cell viability of DU145 and PC3 cells and human dermal fibroblasts were assessed using the SRB assay as followed from previously described protocol (Arjsri et al., 2023). The cells were seeded at 5 × 10^3^ cells per well in 96-well plates and treated with commercially available magnocurarine (MedChemExpress, Monmouth Junction, NJ, United States) at concentrations ranging from 0 to 100 μM for 24 or 48 h. Following treatment, cells were fixed and stained with 100 µL of 0.054% (w/v) SRB solution (Sigma-Aldrich, St. Louis, MO, United States) for 30 min at room temperature. Unbound dye was removed by washing the cells four times with 1% (v/v) acetic acid. Plates were air-dried, and the bound dye was solubilized using 150 µL of 10 mM Tris-base solution (pH 10.5). Absorbance was measured at 510 nm using a microplate reader. Cell viability was expressed as a percentage relative to untreated control cells.

To evaluate cytotoxic effects on red blood cells (RBCs), a hemolysis assay was performed as previously described (Huang et al., 2025). Blood samples were anonymized and handled in accordance with ethical approval (Study code: BIO-2567–0035). Packed RBCs were diluted in 0.86% normal saline solution (NSS) to obtain a 5% (v/v) suspension. Aliquots of 300 µL RBC suspension were incubated with magnocurarine at concentrations ranging from 0 to 10 μM. After incubation, the extent of hemolysis was quantified by measuring the concentration of released hemoglobin, calculated based on a hemoglobin standard calibration curve.

### 2.4 Wound-healing assay for cell migration

Cell migratory capacity was evaluated using a wound-healing (scratch) assay. DU145 and PC3 cells were seeded into 6-well plates at a density of 2.5 × 10^5^ cells per well and cultured for 24 h to form a confluent monolayer. To minimize the influence of cell proliferation on the migration assay, the cells were maintained in DMEM supplemented with 0.5% FBS throughout the treatment period. A uniform scratch was generated across the cell layer using a sterile 200 μL pipette tip. Following wound creation, detached cells were removed, and DMEM supplemented with 0.5% FBS and magnocurarine at concentrations of 0–40 μM was added. Cells were then incubated for an additional 24 h. Images of the same wound regions were captured at 0 and 24 h using a phase-contrast microscope (Nikon Eclipse TS100, Nikon Instruments Inc., Japan) at ×100 magnification.

Wound closure was quantified using the “Wound Healing Size Tool” plugin in ImageJ software (version 1.410) (Suarez-Arnedo et al., 2020). Identical analysis parameters were applied across all experimental groups to ensure consistency in wound area measurement.

### 2.5 Transwell invasion assay

The invasive capacity of CRPC cells was assessed using Transwell chamber inserts fitted with polycarbonate membranes (8 μm pore size; BD Biosciences, Franklin Lakes, NJ, United States). For invasion assays, the upper surface of each insert was pre-coated with 15 μg of Matrigel® (Corning® Basement Membrane Matrix, Cat. No. 356234, Corning Life Sciences, NY, United States) and allowed to polymerize at 37 °C. DU145 and PC3 cells were suspended in serum-reduced DMEM (0.5% FBS) and seeded into the upper chamber at a density of 5 × 10^4^ cells per insert. The lower chamber was filled with DMEM supplemented with 10% FBS to act as a chemoattractant. Cells were treated with magnocurarine (0–40 μM) and incubated for 24 h.

After incubation, non-invading cells were removed, and membranes were gently rinsed with distilled water to eliminate excess staining. Invaded cells were visualized under a phase-contrast microscope (4x), and representative images were acquired. Quantification was performed using the “Threshold” function in ImageJ software (version 1.410), with consistent threshold settings applied across all samples. At least three randomly selected fields per insert were analyzed, and mean values were used for statistical comparison.

### 2.6 Gelatin zymography for MMP activity

Gelatin was incorporated into polyacrylamide gels as a substrate to detect the gelatinolytic activity of MMP-2 (gelatinase A) and MMP-9 (gelatinase B). The cells were seeded in 6-well plates at a density of 1.5 × 10^5^ cells per well and incubated overnight. Cells were pretreated with magnocurarine at the concentration of 0–40 μM for 4 h. The Equal amounts of protein from conditioned media were subjected to electrophoresis on 10% SDS-polyacrylamide gels containing 0.1 mg/mL gelatin under non-reducing conditions. The gels were stained with 0.1% Coomassie Brilliant Blue R-250 (Thermo Fisher Scientific, Rockford, IL, United States). Clear bands of MMP-9 (92 kDa) and MMP-2 (72 kDa) were visualized and quantified using ImageJ software (version 1.410).

### 2.7 Western blot analysis

The expression levels of invasion-related proteins, mesenchymal markers, and MAPK signaling components were determined by Western blot analysis. DU145 and PC3 cells (1.5 × 10^5^ cells per well) were seeded into 6-well plates and treated with magnocurarine (0–40 μM) for 24 h. Cells were then harvested and lysed in RIPA buffer containing protease inhibitors (Thermo Fisher Scientific, Rockford, IL, United States). Protein concentrations were determined using Pierce™ Bradford Plus Protein Assay Reagent (Thermo Fisher Scientific, Waltham, MA, United States). A standard curve was generated using bovine serum albumin as the protein standard. Equal amounts of total protein (30 μg per lane) were separated by 12% SDS–PAGE and transferred onto nitrocellulose membranes. Membranes were incubated overnight at 4 °C with primary antibodies targeting uPA, uPAR, MT1-MMP, N-cadherin, vimentin, phosphorylated ERK (p-ERK), total ERK, phosphorylated JNK (p-JNK), total JNK, phosphorylated p38 (p-p38), and total p38 (Cell Signaling Technology, Danvers, MA, United States), all at a dilution of 1:2000. The membranes were incubated with HRP-conjugated secondary antibodies (Cell Signaling Technology, Danvers, MA, United States) at 1:10,000 ratio of anti-mouse or anti-rabbit IgG for 2 h. The bands were visualized using enhanced chemiluminescence reagents (Cytiva, Marlborough, MA, United States) and captured with the iBright™ CL-1500 imaging system (Thermo Fisher Scientific, Waltham, MA, United States). To verify equal protein loading, membranes were stripped and reprobed with anti-β-actin antibody (Sigma-Aldrich, St. Louis, MO, United States) at 1:10,000 ratio. Band intensities were quantified using ImageJ software (version 1.410). The intensity of each target protein band was normalized to that of the corresponding β-actin band, which served as the loading control. Relative protein expression levels were expressed as percentages, with the normalized value of the untreated control group set to 100%. For phosphorylated proteins, the intensity of each phosphoprotein band was normalized to that of its corresponding total protein band. The resulting phosphoprotein-to-total protein ratios were expressed relative to the untreated control group, for which the normalized value was set to 1.0.

### 2.8 Statistical analysis

All experiments were performed using three independent biological replicates (n = 3), and the data are presented as the mean ± standard deviation (SD). Statistical analyses were performed using one-way analysis of variance (ANOVA), followed by Dunnett’s multiple-comparisons test, with GraphPad Prism version 8.0 (GraphPad Software, San Diego, CA, United States). Statistical significance was defined as *p* < 0.05, *p* < 0.01, or *p* < 0.001.

## 3 Results

### 3.1 Effects of magnocurarine on CRPC cells cytotoxicity

**FIGURE 1:**
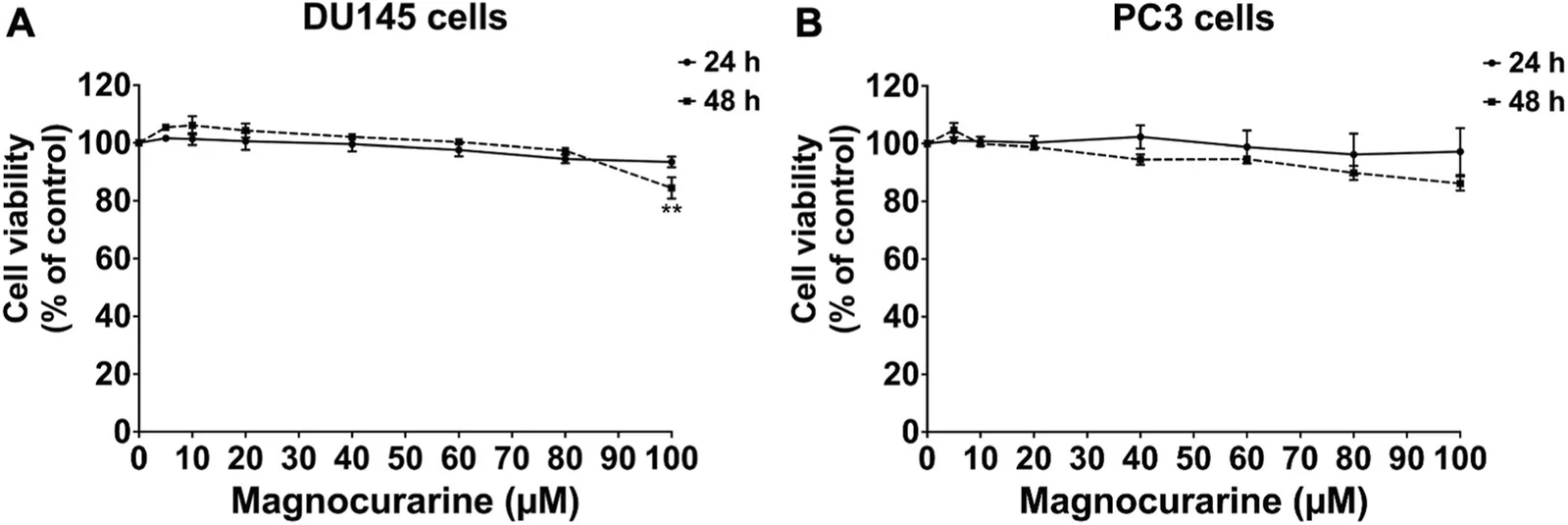
Effects of magnocurarine on cell viability of DU145 **(A)** and PC3 **(B)** prostate cancer cell lines, as determined by the SRB assay (n = 3). **p < 0.01 compared with untreated controls.

Prior to investigating the anti-metastatic effects of magnocurarine, the cytotoxicity against CRPC cells was evaluated to determine appropriate concentrations for subsequent experiments. Cell viability of CRPC cells was assessed using the SRB assay following treatment with increasing concentrations of magnocurarine (0–100 μM) for 24 and 48 h. As shown in Figure 1, magnocurarine did not affect cell viability in either CRPC cell line after 24 h of exposure. At 48 h, a significant reduction in cell viability was observed only in DU145 cells at the highest concentration tested (100 μM) (*p* < 0.01), whereas PC3 cells remained largely unaffected. The calculated IC_50_ values for magnocurarine exceeded 100 μM in both cell lines at both time points, indicating low cytotoxicity within the tested range.

**FIGURE 2:**
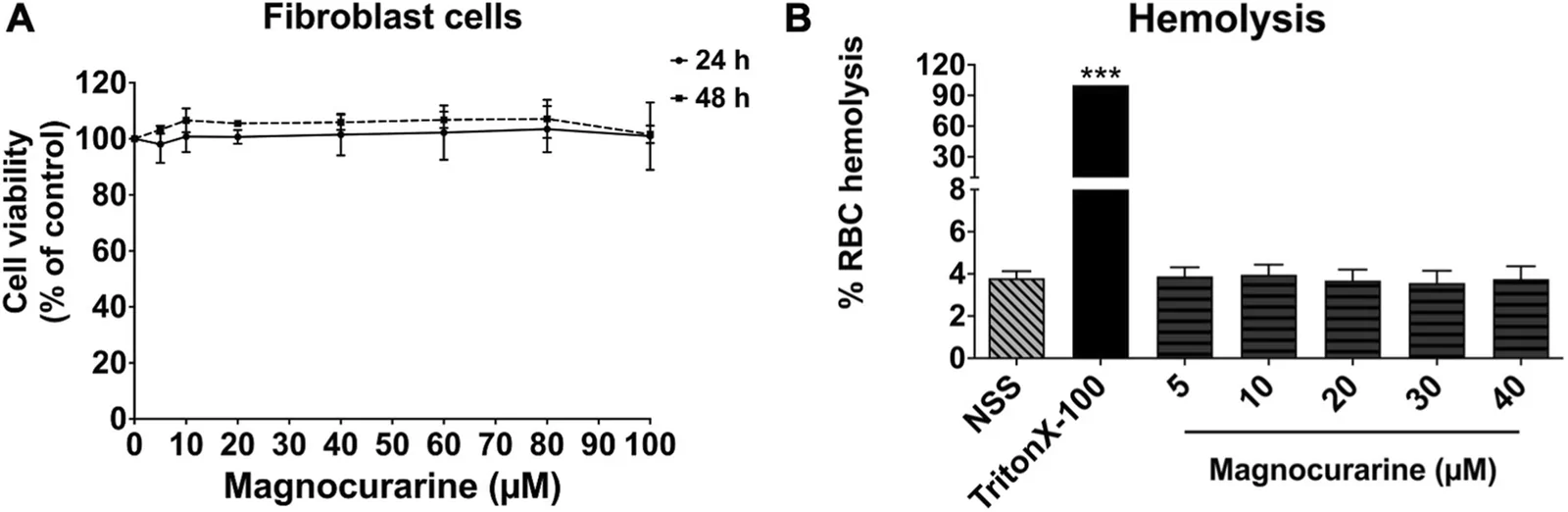
Effects of magnocurarine on the viability of human dermal fibroblasts, as assessed by the SRB assay (n = 3) **(A)**, and on red blood cells, as determined by the hemolysis assay (n = 3) **(B)**. Data are presented as mean ± standard deviation (SD). ***p < 0.001 compared with negative or untreated controls.

Additionally, the cytotoxicity of Magnocurarine was also investigated in normal cells as is shown in Figure 2. The results showed that Magnocurarine displayed significant reduction in %cell viability of human dermal fibroblasts at the concentration >100 μM at both 24 and 48 h. The IC_50_ values of Magnocurarine on human dermal fibroblasts were >100 μM at 24 h and 48 h (Figure 2A). Additionally, Magnocurarine at concentrations from 5–40 μM, did not induce hemolysis in red blood cells (RBCs, Figure 2B). Therefore, based on cytotoxicity profile on CRPC cells and human normal cells, the concentration of 0–40 μM of Magnocurarine were used in the subsequent experiments.

### 3.2 Effects of magnocurarine on migration and invasion of CRPC cells

**FIGURE 3:**
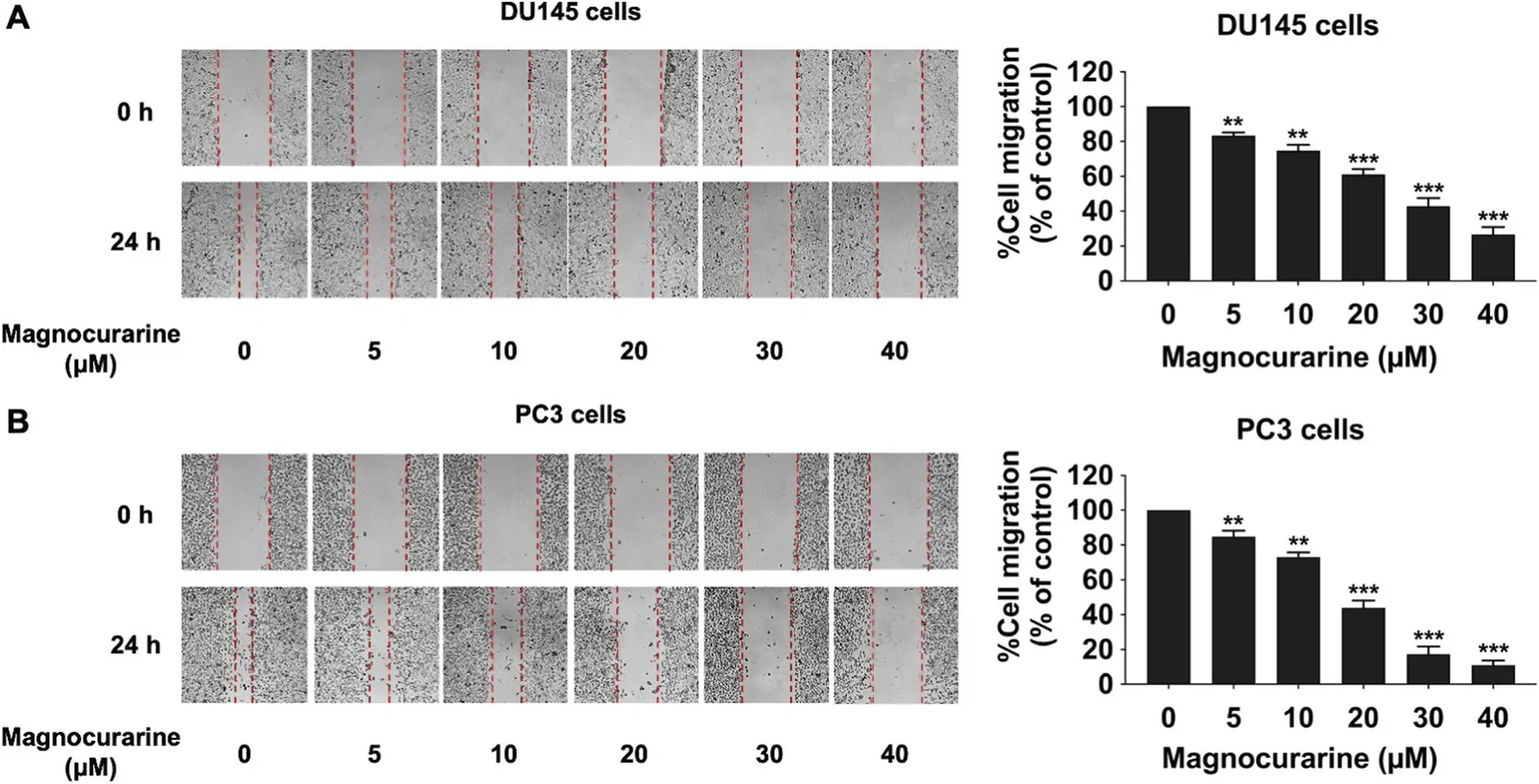
Effects of magnocurarine on CRPC cell migration. Migration of DU145 **(A)** and PC3 **(B)** cells following treatment with magnocurarine (0–40 μM) was evaluated using a wound-healing assay (n = 3). Migration in the untreated control group (0 μM) was set to 100%. Data are presented as mean ± standard deviation (SD). **p < 0.01, and ***p < 0.001 compared with the untreated control group.

The effect of magnocurarine on cell migration was first evaluated using a wound-healing assay. As shown in Figure 3, untreated DU145 (Figure 3A) and PC3 (Figure 3B) cells exhibited substantial wound closure within 24 h, indicating high migratory capacity. In contrast, treatment with magnocurarine significantly reduced wound closure in both cell lines in a concentration-dependent manner (*p* < 0.01), demonstrating an inhibitory effect on cell migration.

**FIGURE 4:**
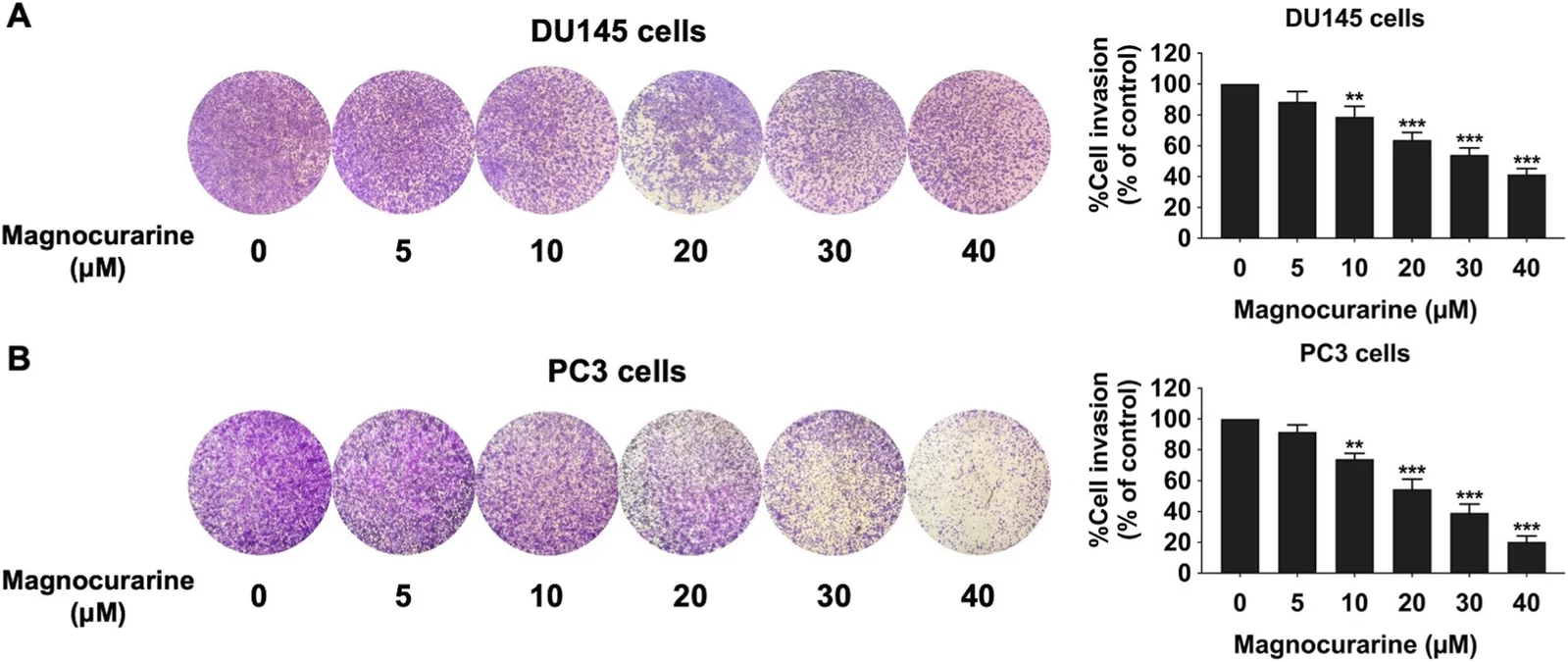
Effects of magnocurarine on CRPC cell invasion. Invasion of DU145 **(A)** and PC3 **(B)** cells following treatment with magnocurarine (0–40 μM) was evaluated using a Transwell invasion assay (n = 3). Invasion in the untreated control group (0 μM) was set to 100%. Data are presented as mean ± standard deviation (SD). **p < 0.01, and ***p < 0.001 compared with the untreated control group.

The anti-invasive activity of magnocurarine was further confirmed using a Transwell invasion assay (Figure 4). Both DU145 (Figure 4A) and PC3 (Figure 4B) cells in the control group displayed pronounced invasive capacity after 24 h. Exposure to magnocurarine resulted in a marked reduction in the number of invading cells in both cell lines, with a clear concentration-dependent trend (*p* < 0.01). Together, these results indicate that magnocurarine effectively suppresses both migratory and invasive behaviors of CRPC cells.

### 3.3 Effects of magnocurarine on MMP-2 and MMP-9 activities and invasion-related protein expression in CRPC cells

**FIGURE 5:**
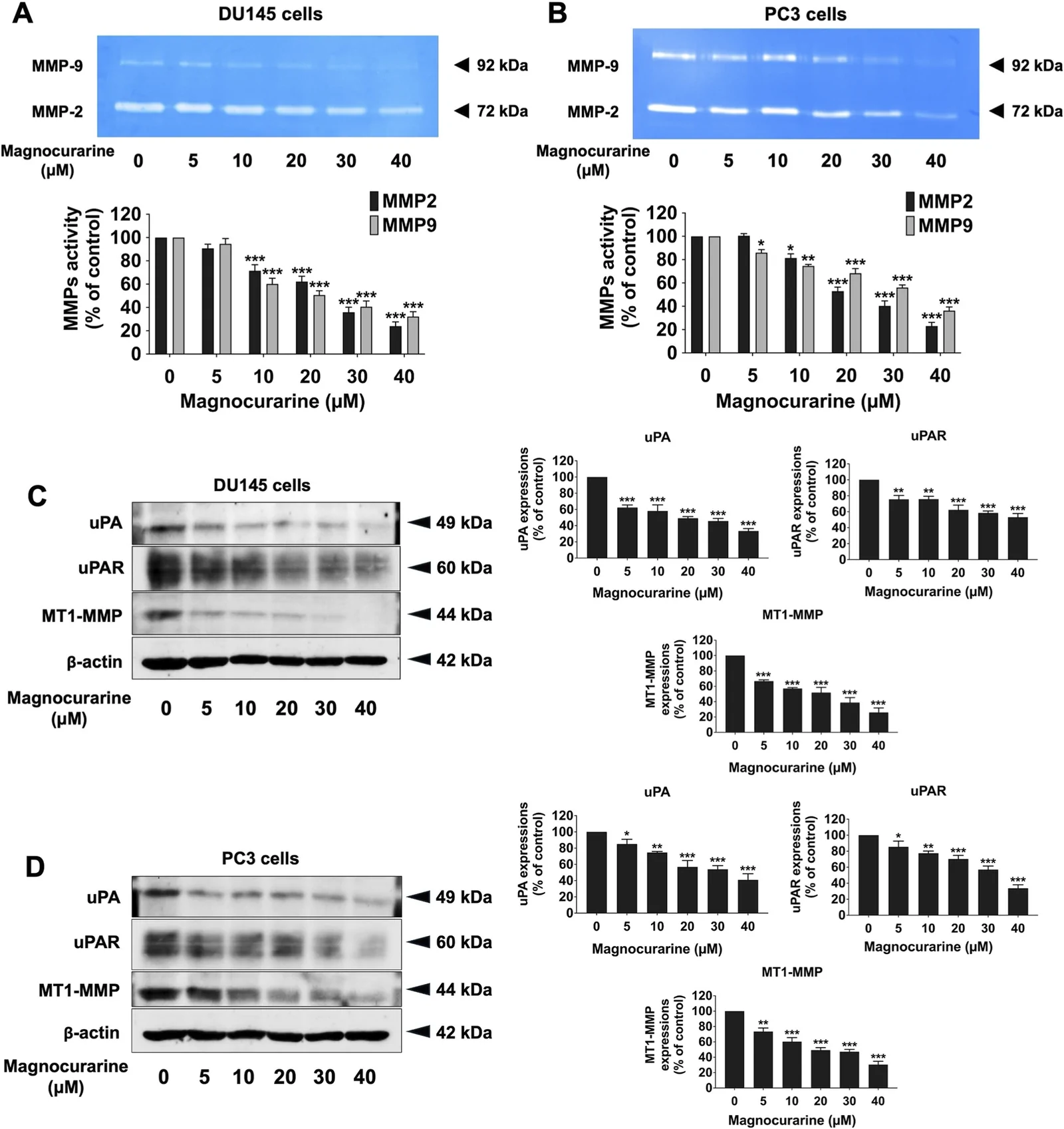
Effects of magnocurarine on MMP activity and invasion-related protein expression in CRPC cells. The inhibitory effects of magnocurarine on MMP-9 (92 kDa) and MMP-2 (72 kDa) activity in DU145 **(A)** and PC3 **(B)** cells were assessed by gelatin zymography (n = 3). The expression levels of uPAR (60 kDa), uPA (49 kDa), and MT1-MMP (44 kDa) in DU145 **(C)** and PC3 **(D)** cells were determined by Western blot analysis (n = 3). Quantitative densitometric analysis of uPAR, uPA, and MT1-MMP was normalized to β-actin (42 kDa) as the loading control. Data are presented as mean ± standard deviation (SD). *p < 0.05, **p < 0.01, and ***p < 0.001 compared with the untreated control group.

The effects of magnocurarine on matrix metalloproteinase activity were examined by gelatin zymography. As shown in Figures 5A,B, prominent gelatinolytic bands corresponding to MMP-9 (92 kDa) and MMP-2 (72 kDa) were observed in control CRPC cells. Treatment with magnocurarine significantly reduced the activity of both MMP-2 and MMP-9 in DU145 and PC3 cells in a concentration-dependent manner (*p* < 0.05). To further investigate the molecular basis of reduced invasiveness, the expression of inva-sion-associated proteins was analyzed by Western blotting. Magnocurarine treatment resulted in a substantial decrease in the expression levels of uPA, uPAR, and MT1-MMP in both CRPC cell lines (Figures 5C,D; p < 0.05). These findings suggest that magnocurarine attenuates invasive phenotypes in CRPC cells by suppressing both MMP activity and key proteolytic regulators involved in extracellular matrix degradation.

### 3.4 Effects of magnocurarine on mesenchymal marker expression in CRPC cells

**FIGURE 6:**
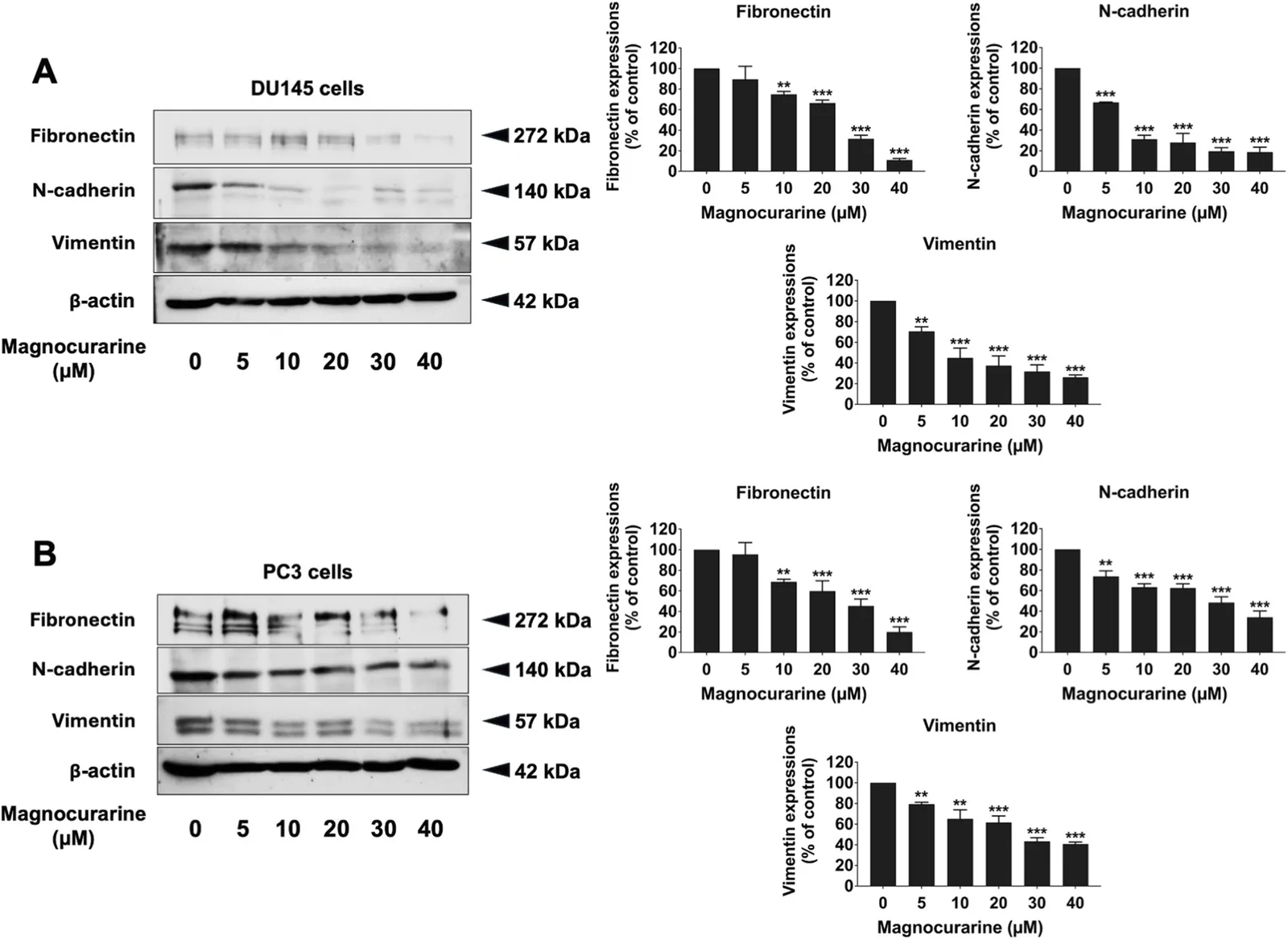
Effects of magnocurarine on mesenchymal marker expression in CRPC cells. The expression levels of fibronectin (272 kDa), N-cadherin (140 kDa), and vimentin (57 kDa) in DU145 **(A)** and PC3 **(B)** cells were determined by Western blot analysis (n = 3). Quantitative densitometric analysis of mesenchymal markers were normalized to β-actin (42 kDa) as the loading control. Data are presented as mean ± standard deviation (SD). **p < 0.01, and ***p < 0.001 compared with the untreated control group.

To determine whether magnocurarine affects the expression of mesenchymal markers, the levels of N-cadherin, vimentin, and fibronectin were evaluated. As shown in Figure 6, treatment with magnocurarine significantly reduced the expression of N-cadherin, vimentin, and fibronectin in both DU145 (Figure 6A) and PC3 (Figure 6B) cells (*p* < 0.01). The observed downregulation of these mesenchymal markers suggests that magnocurarine suppresses the mesenchymal phenotype, which is closely associated with enhanced migration and invasion in cancer cells.

### 3.5 Effects of magnocurarine on MAPK signaling in CRPC cells

**FIGURE 7:**
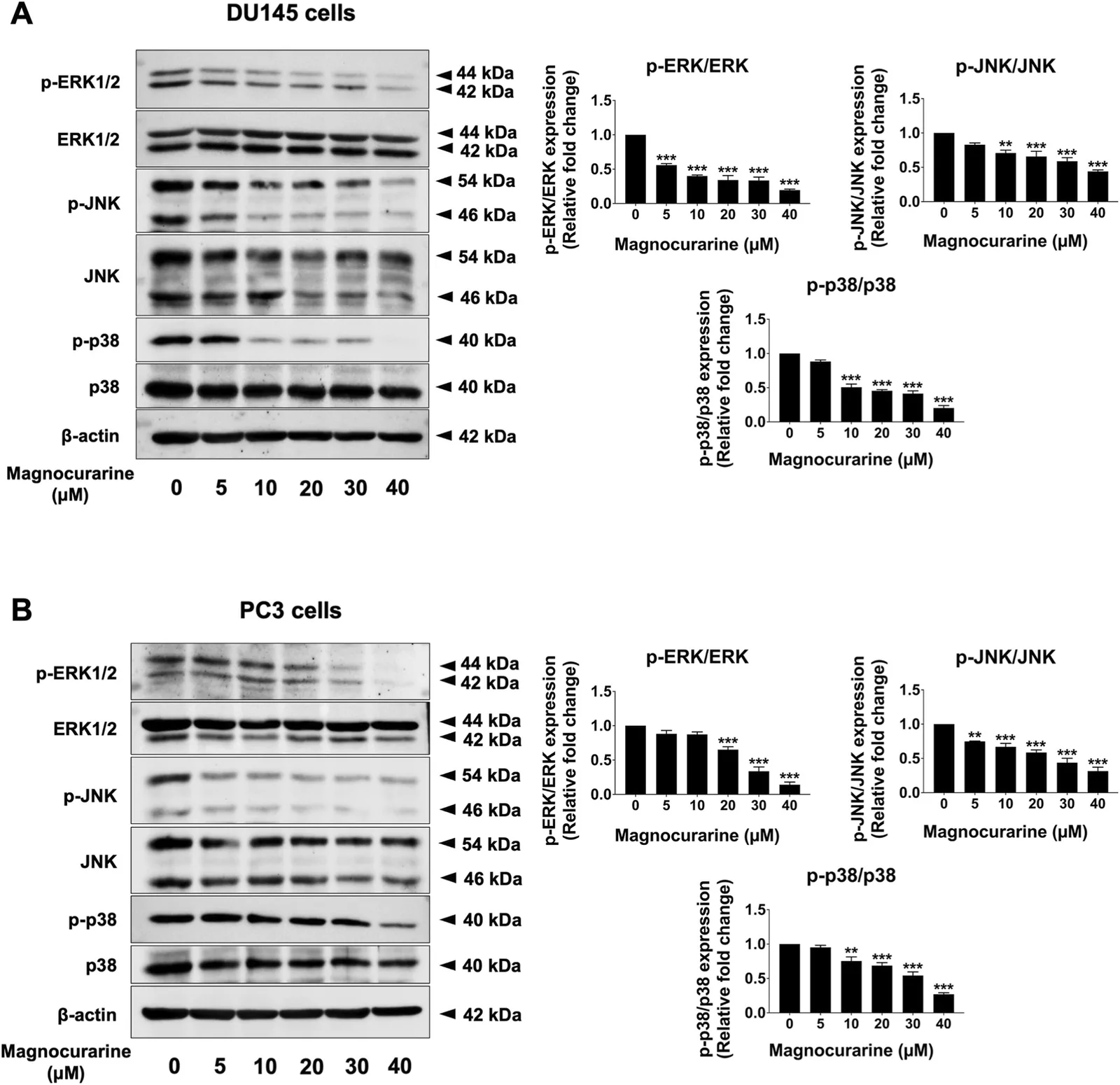
Effects of magnocurarine on MAPK signaling in CRPC cells. The phosphorylation levels of ERK, JNK, and p38 in DU145 **(A)** and PC3 **(B)** cells were analyzed by Western blot (n = 3), along with corresponding densitometric quantification. Phosphorylated protein levels were normalized to their respective total ERK, JNK, or p38 levels and expressed relative to the untreated control group (set as 1.0). Data are presented as mean ± standard deviation (SD). **p < 0.01 and ***p < 0.001 compared with the untreated control group.

To elucidate the underlying molecular mechanism, the effect of magnocurarine on MAPK signaling was examined by Western blot analysis. As shown in Figure 7, untreated CRPC cells exhibited elevated levels of phosphorylated ERK, JNK, and p38, indicating activation of MAPK signaling pathways. Treatment with magnocurarine significantly decreased the phosphorylation levels of ERK, JNK, and p38 in both DU145 (Figure 7A) and PC3 (Figure 7B) cells in a concentration-dependent manner (*p* < 0.05), while the total protein levels remained unchanged. These results demonstrate that magnocurarine selectively inhibits MAPK pathway activation, which may contribute to its suppressive effects on migration and invasion in CRPC cells.

## 4 Discussion

**FIGURE 8:**
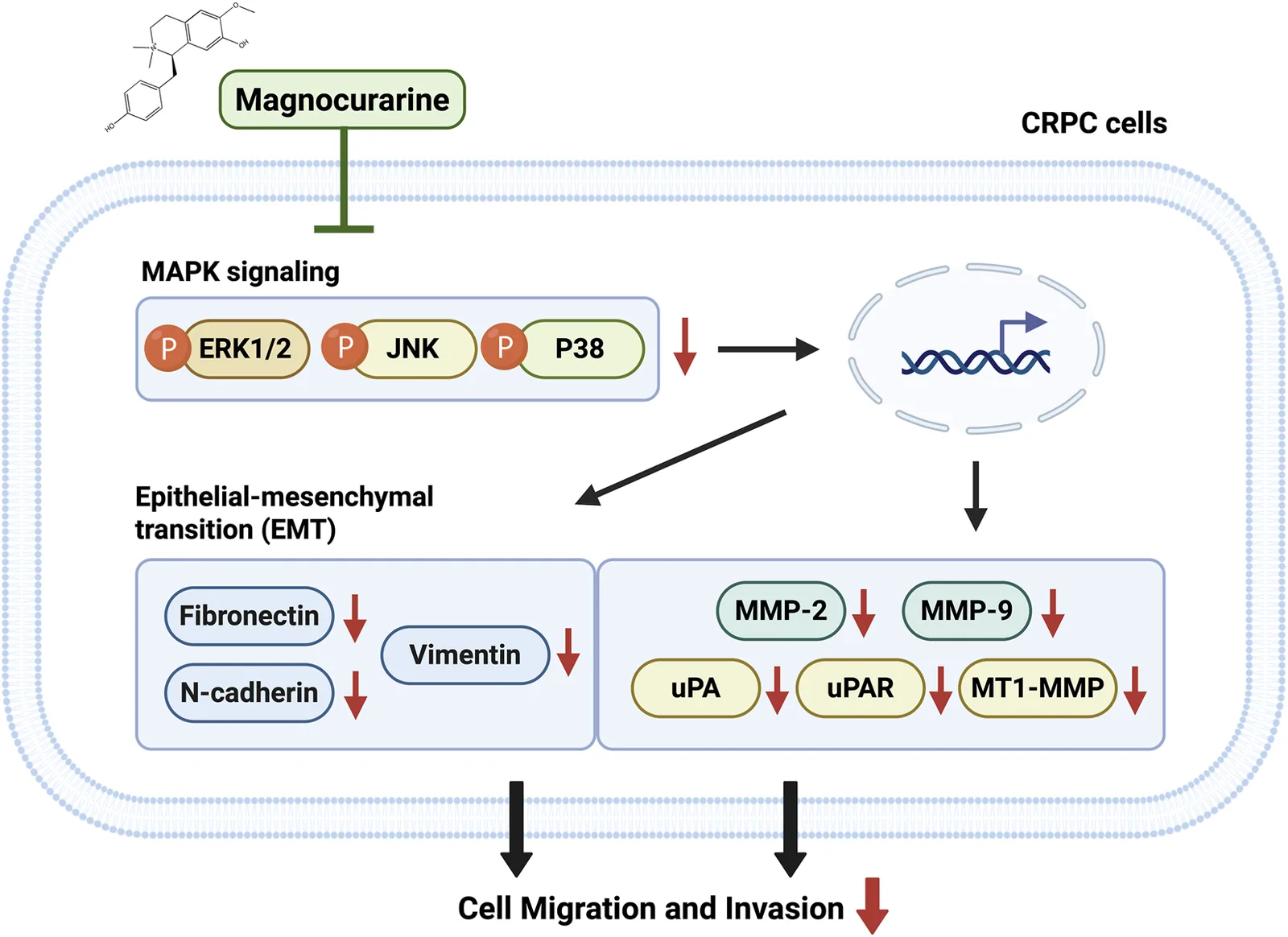
The mechanism of magnocurarine interferes with pro-metastatic signaling cascades in CRPC cells.

In the present study, magnocurarine suppressed the migratory and invasive properties of CRPC cells and modulated MAPK signaling. Specifically, magnocurarine inhibited cell migration and invasion, reduced MMP activities, and downregulated key invasion-associated proteins and mesenchymal markers, including N-cadherin, vimentin, and fibronectin. Magnocurarine also decreased the phosphorylation levels of ERK, JNK, and p38, indicating suppression of MAPK pathway activation. Collectively, these results suggest that magnocurarine interferes with pro-metastatic features and MAPK signaling cascades in CRPC cells. The proposed mechanism is illustrated in Figure 8.

CRPC is characterized by highly metastatic phenotypes and is often regarded as a lethal type of prostate cancer due to its poor prognosis to conventional chemotherapy (Raychaudhuri et al., 2025; Schafer et al., 2025). Current treatment strategy for CRPCs is based on patient status, comorbidities, tumor stage, and importantly the molecular characteristics of cancer. Identification of targetable mutations has revealed multiple dysregulated signaling pathways associated with the pathogenesis of prostate cancer, particularly those involved in cell migration, invasion, and metastatic progression. These pathways include EGFR, PI3K, mTOR, and MAPK (Alobid, 2026; Jadvar and Iravani, 2026). Recent phosphor-proteomic analysis of CRPC tumors have revealed extensive activation of multiple druggable kinases within MAPK pathway, suggesting that aberrant signaling activity drives oncogenic processes in CRPC. Furthermore, kinase activation profiles tend to remain consistent across anatomically distinct metastatic lesions within the same patient, despite the heterogeneity between patients (Morgan et al., 2009; Drake et al., 2013).

In this study, we investigate magnocurarine as a potential therapeutic candidate for CRPC. We found that magnocurarine inhibited CRPC cancer cell migration and invasion in two CRPC cell lines, DU145 and PC3. Additionally, magnocurarine suppressed the ERK/JNK/p38 MAPK signaling via inhibiting the phosphorylation of ERK, JNK, and p38 proteins. To our knowledge, this is the first study reporting the anti-migratory and anti-invasion effects of the magnocurarine on CRPC cells. This study provides mechanistic evidence that magnocurarine derived from Magnoliaceous plants suppresses the activation of the MAPK pathway in CRPC. These findings suggest that magnocurarine may affect signaling pathways involved in prostate cancer cell migration and invasion, supporting the potential role of phytochemical compounds in modulating cancer progression-associated phenotypes (Meirson et al., 2020). However, the present study did not identify the direct molecular targets of magnocurarine, such as specific receptors or upstream kinases. Further studies using MAPK pathway-specific inhibitors, and siRNA-mediated knockdown approaches are required to elucidate the precise molecular mechanisms involved and to determine whether MAPK signaling is a key mediator of the anti-migratory and anti-invasive effects of magnocurarine.

Our study investigated the anticancer progression and anti-invasion properties of magnocurarine on two representative CRPC cell lines. The selection of these two cell lines was based on the investigation on the inhibition of cancer cells progression properties of our candidate phytochemical on the MAPK signaling in CRPCs (Mukherjee et al., 2011; Nickols et al., 2019). Previous studies found that inhibition of JNK in DU145 cells reduced both cell migration and the expression of VEGF protein, which is involved in the prostate cancer growth and metastasis (Tyagi et al., 2003). Additionally, the p38 MAPK signaling is essential for TGF-mediated MMP-2 activity, and thus, the invasion in PC3 cancer cells (Mukhopadhyay et al., 2006). While p38 in DU145 cells contributed to the invasion and migration capabilities by enhancing the MMP-2 and MMP-9 activities, and u-PA expression (Shen et al., 2010). Therefore, these two cell lines are widely used models to represent the aggressiveness of prostate cancer with the association of MAPK signaling activation.

Although the anticancer effects of magnocurarine have received limited attention, magnoflorine, a structurally related but distinct quaternary aporphine alkaloid that shares a benzylisoquinoline biosynthetic origin with magnocurarine, has been reported to possess anticancer properties. In MDA-MB-468 breast cancer, NCI-H1299 lung cancer, T98G glioma, and TE671 rhabdomyosarcoma cell lines, magnoflorine inhibited cell proliferation, induced caspase-3–dependent apoptosis, and arrested the cell cycle at the S/G2 phase in a dose-dependent manner, while exhibiting comparatively low cytotoxicity toward normal fibroblasts (Okon et al., 2020; Xu et al., 2020). In breast cancer models, magnoflorine also enhanced the anti-proliferative, anti-migratory, and pro-apoptotic effects of doxorubicin by suppressing PI3K/AKT/mTOR signaling and promoting p38 MAPK–dependent apoptosis and autophagy (Wei et al., 2020). These findings suggest that structurally related aporphine and benzylisoquinoline alkaloids may interact with cancer-associated signaling pathways, including MAPK signaling. However, the direction of MAPK modulation may be compound- and context-dependent. Whereas magnoflorine activated p38 MAPK in association with doxorubicin-induced apoptosis in breast cancer cells, magnocurarine reduced p38 phosphorylation in the CRPC cell models examined in the present study. Further mechanistic studies are required to determine whether these distinct effects are attributable to differences in compound structure, cancer type, or experimental context.

Magnocurarine selectively reduced the phosphorylation of MAPK signaling proteins without affecting their total expression levels, indicating targeted inhibition of pathway activation. These findings are consistent with previous studies demonstrating that inhibition of MAPK signaling leads to suppression of metastatic phenotypes in prostate cancer cells (Mukherjee et al., 2011; Cheung et al., 2021). Given that MAPK signaling regulates the transcription of genes associated with invasion and metastasis, including MMPs, components of the urokinase system, and EMT-related markers (Santibanez et al., 2018; Papanikolaou et al., 2021), inhibition of MAPK signaling provides a mechanistic explanation for the reductions in gelatinolytic activity and the responsible protein expressions. The coordinated downregulation of MMP-2, MMP-9, uPA/uPAR/MT1-MMP, and mesenchymal markers further support the anti-invasive and anti-metastatic effects of magnocurarine. In addition to its anti-metastatic activity, magnocurarine exhibited low cytotoxicity in normal human dermal fibroblasts and in red blood cells at the concentrations tested. These findings suggest a favorable safety profiles for magnocurarine and support the potential for their use in therapeutic applications without severe adverse effects on normal cells.

The relevance of MAPK signaling to the present findings is further supported by its established role in CRPC progression and treatment resistance. Transcriptomic and whole-genome analyses of metastatic CRPC have identified hyperactive ERK1 and recurrent amplification of MAPK pathway genes in approximately one-third of tumors, and phosphorylated ERK1/2 is elevated in CRPC relative to untreated primary prostate cancer. Because ERK is activated downstream of MEK1/2, this pathway represents a potentially actionable therapeutic target. Indeed, the MEK inhibitor trametinib has produced biochemical and clinical responses in heavily pretreated patients with metastatic CRPC (Nickols et al., 2019). MAPK signaling has also been implicated in resistance to androgen receptor-targeted therapy. Following enzalutamide-mediated AR inhibition, the atypical chemokine receptor CXCR7 is derepressed and activates ERK through a ligand-independent, β-arrestin 2-dependent mechanism. The expression of CXCR7 and phosphorylated ERK progressively increases from localized prostate cancer to CRPC and further increases following the development of enzalutamide resistance. Conversely, inhibition of MAPK/ERK signaling markedly suppresses the growth of resistant tumors (Li et al., 2019). Furthermore, p38 MAPK has been reported to sustain AR activity under hypoxic conditions in CRPC, while inhibition of BRAF/MEK/ERK signaling may restore enzalutamide sensitivity in genetically susceptible tumors. Collectively, these observations indicate that MAPK activation may contribute to CRPC progression and may provide a bypass mechanism that enables resistance to AR blockade. These findings provide a biological rationale for further investigating the association between MAPK modulation and the anti-migratory and anti-invasive effects of magnocurarine. However, the present results do not establish a direct causal relationship between MAPK suppression and the observed cellular effects.

In conclusion, magnocurarine suppressed migration and invasion of CRPC cells, accompanied by the downregulation of MMPs, invasive-associated proteins, and mesenchymal markers. Magnocurarine treatment was also associated with reduced phosphorylation of ERK1/2, JNK1/2, and p38 in both CRPC cell lines, indicating modulation of MAPK signaling. However, because the present study did not include mechanistic rescue experiments, these findings establish an association rather than a direct causal relationship between MAPK pathway suppression and the anti-migratory and anti-invasive effects of magnocurarine. These results suggest that magnocurarine may have potential as a therapeutic candidate for advanced prostate cancer; however, further investigation is required before its therapeutic relevance can be established. Several limitations of the present study should be acknowledged. First, all experiments were conducted using *in vitro* CRPC cell models; therefore, the observed effects on cell migration and invasion cannot be considered equivalent to the inhibition of metastasis *in vivo*. Second, although magnocurarine treatment was associated with decreased phosphorylation of ERK1/2, JNK1/2, and p38, mechanistic rescue experiments using MAPK activators, pathway-specific inhibitors, or siRNA-mediated knockdown approaches were not performed. Consequently, the present findings do not establish whether MAPK signaling directly mediates the anti-migratory and anti-invasive effects of magnocurarine. Third, the direct molecular targets and upstream regulators affected by magnocurarine remain unidentified. Finally, the pharmacokinetic properties, safety, antitumor activity, and antimetastatic effects of magnocurarine have not yet been evaluated in animal models. Further mechanistic, *in vivo*, and preclinical studies are therefore required to clarify its molecular targets and therapeutic potential, including its possible synergistic effects with existing therapeutic agents (Quinn et al., 2017; Gu et al., 2025). These investigations should be accompanied by detailed molecular studies to clarify the underlying mechanisms of action.

## Data Availability

The raw data supporting the conclusions of this article will be made available by the authors, without undue reservation.
